# Preseason Screening Tests and Physical Assessments as Predictors of Injury in Handball Players: A Systematic Review

**DOI:** 10.3390/sports14060234

**Published:** 2026-06-05

**Authors:** Stelios Hadjisavvas, Irene-Chrysovalanto Themistocleous, Elena Papamichael, Michalis A. Efstathiou, Christina Michailidou, Manos Stefanakis

**Affiliations:** Department of Health Sciences, University of Nicosia, CY-2417 Nicosia, Cyprus; themistocleous.i@unic.ac.cy (I.-C.T.); papamichael.el@unic.ac.cy (E.P.); efstathiou.m@unic.ac.cy (M.A.E.); michailidou.c@unic.ac.cy (C.M.); stefanakis.m@unic.ac.cy (M.S.)

**Keywords:** handball, injury outcomes, preseason screening, shoulder strength, range of motion, systematic review

## Abstract

Background: Preseason screening is widely used in handball to identify athletes at increased risk of injury, yet the prognostic value of different screening approaches remains unclear. The aim of this study was to systematically review the evidence on preseason screening tests and physical assessments in relation to subsequent injury outcomes in handball players. Methods: A systematic review was conducted according to PRISMA guidelines. PubMed, MEDLINE, CINAHL, and Scopus were searched on 14 March 2026. The first 100 results from Google Scholar were also screened, and backward citation searching was performed. Eligible studies included handball players and examined preseason or baseline screening, functional, musculoskeletal, or physical performance assessments in relation to prospectively recorded injury outcomes. Two independent reviewers performed study selection, data extraction, and risk-of-bias assessment using the QUIPS tool. Due to substantial heterogeneity in screening tools, injury outcomes, and follow-up procedures, meta-analysis was not performed. Results: Eight studies were included. Most were prospective cohorts involving adolescent, youth elite, or elite adult handball players. Shoulder-specific screening variables, particularly external rotation strength, strength imbalances, total rotational motion, and selected rotational adaptations, showed more consistent associations with subsequent shoulder-related outcomes. In contrast, broader movement-screening tools, including the Functional Movement Screen, the 9+ screening battery, and the upper quarter Y-Balance Test, generally showed limited associations with overall injury outcomes. Conclusions: Shoulder-specific preseason assessments may be more closely associated with subsequent shoulder-related outcomes than broader movement-screening tools, although the available evidence remains limited, heterogeneous, and derived exclusively from observational studies.

## 1. Introduction

Handball is a physically demanding intermittent team sport that involves repeated accelerations, changes of direction, jumping, overhead throwing, and frequent player contact. As a result, the sport is associated with a substantial injury burden. Although lower-limb injuries are the most common overall, the shoulder is the most frequently affected upper-extremity region, and overuse problems—particularly at the shoulder and knee—have a meaningful impact on training participation and performance [1,2]. Furthermore, shoulder problems appear to be particularly relevant in adolescent and elite handball populations, where repetitive throwing exposure is high and substantial shoulder problems are commonly reported [3].

In this context, preseason or baseline screening is commonly used to identify athletes who may be at increased risk of subsequent injury and to guide preventive interventions. In overhead sports more broadly, factors such as shoulder range of motion, rotator cuff weakness, and training load have been associated with subsequent shoulder injury risk. However, this evidence is heterogeneous and cannot be directly extrapolated to handball, as the sport combines repetitive throwing with contact exposure, positional demands, and a distinct injury profile. Importantly, a previous systematic review and meta-analysis in overhead athletes suggested that preseason shoulder range-of-motion screening may have limited value for identifying handball players at risk of injury, highlighting the need for sport-specific synthesis rather than extrapolation from mixed-sport evidence [4,5].

Although the number of handball-specific studies investigating potential prognostic screening variables has increased, the available evidence synthesis remains incomplete. Previous systematic reviews in handball have primarily focused on injury epidemiology and shoulder injury risk factors [2,6]. However, to our knowledge, no systematic review has specifically synthesized the evidence regarding preseason screening and physical assessment procedures used in relation to prospectively recorded injury outcomes in handball players. In particular, it remains unclear whether sport-specific shoulder assessments and broader movement-screening tools demonstrate consistent prognostic associations with subsequent injury outcomes in this population. This is an important gap, because screening in handball is not limited to shoulder-specific assessments; it also includes broader movement-screening tools and functional performance tests. Furthermore, it remains unclear whether different screening approaches provide distinct prognostic information regarding subsequent injury outcomes in handball players. Accordingly, the aim of this systematic review was to synthesize the available evidence on preseason or baseline screening tests and physical assessments in relation to subsequent injury outcomes in handball players.

## 2. Methods

### 2.1. Study Design

This systematic review was conducted and reported in accordance with the Preferred Reporting Items for Systematic Reviews and Meta-Analyses (PRISMA 2020) statement [7]. The review protocol was prospectively registered in PROSPERO (CRD420261340712) and is publicly available through the PROSPERO database. No major protocol amendments were made after registration.

### 2.2. Eligibility Criteria

Eligibility criteria were defined according to the PICOS framework. The population of interest included male and female handball players of any competitive level and age category. Studies were considered eligible when they examined preseason or baseline screening assessments, including functional movement tests, musculoskeletal tests, strength measures, range of motion assessments, balance tests, or other physical performance tests in relation to subsequent injury outcomes. Because this review focused on prognostic screening variables rather than interventions, the presence of a comparator group was not required for study inclusion. The outcome of interest was any subsequent sports-related injury, including time-loss injuries, overuse injuries, shoulder injuries, lower-limb injuries, or other musculoskeletal injuries recorded prospectively during follow-up. Regarding study design, only original prospective or longitudinal observational studies were included. Reviews, systematic reviews, meta-analyses, case reports, conference abstracts, letters, book chapters, and methodological or reliability studies without injury follow-up were excluded. Studies were also excluded if they were not handball-specific and did not provide extractable data for handball players, or if they did not assess baseline screening measures in relation to later injury risk. Broad eligibility criteria were intentionally applied because preseason screening in handball includes a wide range of approaches, including shoulder-specific assessments, movement-screening tools, balance tests, and general physical performance measures. The aim of the review was therefore to synthesize the available evidence regarding prognostic screening variables across the spectrum of preseason screening procedures used in handball settings.

### 2.3. Information Sources and Search Strategy

A systematic literature search was conducted on 14 March 2026 in PubMed, MEDLINE, CINAHL, and Scopus. In addition, Google Scholar was used as a supplementary search source. Results were sorted by relevance, and the first 100 records were screened for potential eligibility, consistent with common systematic review practice. In addition, backward citation searching of relevant reviews and included studies was performed to identify additional eligible articles. The search strategy combined terms related to handball, screening or functional testing, and injury risk. A core search string was developed and adapted to the syntax of each database. The complete database-specific search strategies, database platforms, Boolean operators, and supplementary search procedures are provided in Supplementary File S1 in accordance with PRISMA 2020 recommendations [7].

### 2.4. Study Selection

All records identified through database searching, Google Scholar screening, and citation searching were imported into a single dataset and screened after duplicate removal. Study selection was performed in two stages. First, two independent reviewers screened the titles and abstracts of all retrieved records for potential relevance. Second, the full texts of potentially eligible studies were assessed independently by the same two reviewers against the predefined eligibility criteria. Any disagreements between the two reviewers were resolved through discussion; when consensus could not be reached, a third reviewer was consulted. Reasons for exclusion were documented at both the title/abstract screening stage and the full-text stage. The overall study selection process was documented using a PRISMA flow diagram [7].

### 2.5. Data Extraction

Data were extracted independently by two reviewers (SH and ICT) using a standardized data-extraction form. The extracted information included first author, publication year, study design, sample characteristics, screening or baseline assessment, injury outcome, follow-up duration, and the main findings related to injury risk. Any discrepancies in data extraction were resolved through discussion, with consultation of a third reviewer (EP) when necessary. Data extraction additionally included reported effect estimates (e.g., ORs, HRs, RRs, AUC values), whether estimates were adjusted or unadjusted, confounding variables considered in the original analyses, number of injury events, and reported exposure or follow-up data when available.

### 2.6. Risk of Bias Assessment

Risk of bias was assessed independently by two reviewers (SH and MAE) using the Quality In Prognosis Studies (QUIPS) tool, which is recommended for studies examining prognostic factors [8]. The following domains were evaluated: study participation, study attrition, prognostic factor measurement, outcome measurement, confounding, and statistical analysis and reporting. Any disagreements were resolved through discussion; when agreement could not be reached, a third reviewer (MS) was consulted [8]. Overall study-level risk-of-bias judgments were derived through reviewer consensus based on the combination of domain-level QUIPS ratings. Studies with multiple moderate-risk domains or at least one high-risk domain were judged as having moderate-to-high overall risk of bias.

### 2.7. Data Synthesis

Due to substantial heterogeneity across the included studies in terms of screening or baseline assessments, injury definitions, outcome measures, and follow-up procedures, a meta-analysis was not considered appropriate. Therefore, a narrative synthesis was undertaken. Formal assessment of reporting bias and certainty of evidence was not performed because of the small number of included studies, substantial methodological heterogeneity, and the absence of sufficiently comparable outcome measures to support quantitative synthesis. The results were grouped according to the type of screening or baseline assessment, including functional movement tests, balance tests, shoulder range of motion, strength measures, and other shoulder-related musculoskeletal factors. When available, the direction and magnitude of associations between baseline measures and subsequent injury outcomes were summarized. Because of heterogeneity in study design, screening procedures, outcome definitions, and statistical methods, effect estimates including odds ratios (ORs), hazard ratios (HRs), relative risks (RRs), and area under the curve (AUC) values were synthesized narratively rather than quantitatively pooled.

## 3. Results

### 3.1. Results of the Study Selection Process

**Figure 1:**
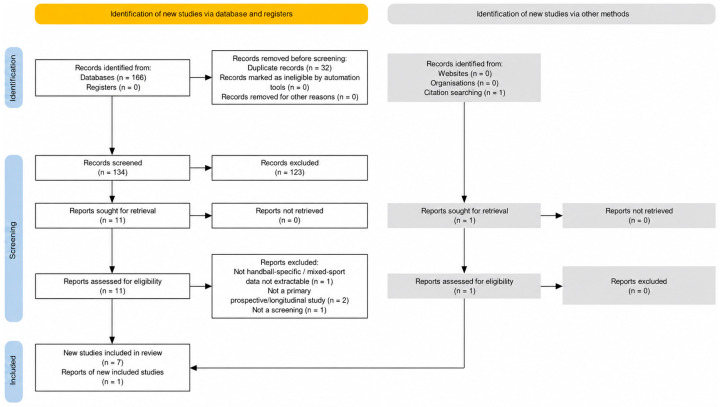
PRISMA flow diagram of the included studies.

A total of 166 records were identified through database and supplementary searching. After removal of 32 duplicates, 134 records remained for title and abstract screening. Of these, 123 were excluded. Eleven full-text articles were assessed for eligibility, and seven studies met the inclusion criteria. Following backward citation searching, one additional eligible study was identified and included. Overall, eight studies were included in the review. The study selection process is presented in the PRISMA flow diagram (Figure 1).

### 3.2. Study Characteristics

#### 3.2.1. Overview of the Included Studies

Eight studies were included in this review, comprising a total sample of 1155 handball players. Most studies used a prospective cohort design, whereas one study adopted a mixed design with retrospective injury history assessment and a 6-month follow-up after screening [9,10,11,12,13,14,15,16]. The included literature was geographically concentrated in Europe, with studies conducted in Germany, Norway, Sweden, France, and Poland, and most cohorts involved highly trained adolescent or elite adult handball players. The main characteristics of the included studies are presented in Table 1.

#### 3.2.2. Study Design and Settings

The included studies were conducted in Germany, Norway, Sweden, France, and Poland. Seven studies followed players prospectively across one or more competitive seasons, allowing baseline screening measures to be examined in relation to subsequent injury outcomes. One study, however, employed a less robust mixed design involving retrospective injury-history collection and subsequent self-reported follow-up, and its findings should therefore be interpreted more cautiously with respect to prognostic inference [16]. In general, the study settings were sport-specific and involved organized handball environments, including national handball high schools, elite league teams, federal state squads, and national-team cohorts.

#### 3.2.3. Participant Characteristics

The study populations were heterogeneous with respect to age, sex, and competitive level. Four studies focused on adolescent players [9,10,12,15], whereas the remaining studies included adult elite or high-level handball players [11,13,16], or youth elite female national-team players close to adulthood [14]. Sex distribution also varied across the included studies. One study included only male players [13], one included only female players [14], and several studies involved mixed-sex cohorts [9,10,11,15]. The full-text article by Bauer et al. [12] was reviewed in detail. Minor inconsistencies in sex distribution reporting were identified between the abstract and baseline characteristics table. For the purposes of this review, the baseline characteristics table reported in the full-text article was used as the primary source of participant information.

#### 3.2.4. Types of Screening or Baseline Assessment

A clear distinction emerged between studies that examined shoulder-specific preseason screening and those that evaluated broader movement-screening tools. Five studies focused specifically on shoulder-related musculoskeletal or performance measures, including glenohumeral range of motion, total rotational motion, humeral retrotorsion, isometric or isokinetic rotator cuff strength, strength ratios, and scapular dyskinesis [9,10,11,13,14]. These studies focused on screening variables directly related to the repetitive throwing demands of handball and mainly examined measures of the dominant shoulder.

In contrast, three studies investigated broader movement-screening tools. Karlsson et al. [15] assessed the 9+ screening battery, Slodownik et al. [16] used the Functional Movement Screen, and Bauer et al. [12] examined upper quarter Y-balance test performance and asymmetry. Compared with the shoulder-focused studies, these investigations reflected broader movement constructs and were less directly linked to a specific anatomical region or injury mechanism.

#### 3.2.5. Injury Outcomes and Follow-Up Procedures

The included studies also differed in their injury outcomes and surveillance procedures. Five studies focused on shoulder-specific injury outcomes, particularly overuse shoulder problems or subsequent shoulder injuries in the dominant arm [9,10,11,13,14]. These studies typically used prospective surveillance through repeated questionnaires or physician-led injury recording and were more closely aligned with the throwing demands of handball.

The remaining studies examined broader outcomes such as any sport-related injury, non-contact injury, substantial injury, or time-loss injury during follow-up [12,15,16]. Follow-up duration ranged from six months to two seasons, and the frequency of surveillance varied considerably, from weekly monitoring to repeated assessments at predefined seasonal time points or monthly physician follow-up. This heterogeneity in outcome definition and monitoring likely affected comparability across studies and was one of the main reasons a meta-analysis was not considered appropriate.

#### 3.2.6. Outcome Definitions and Injury Assessment

The included studies differed considerably in how subsequent injury outcomes were defined and assessed. Shoulder-specific studies commonly used patient-reported overuse measures, including WOSI-based surveillance in the studies by Achenbach et al. [9,10] and the OSTRC Overuse Injury Questionnaire in the studies by Clarsen et al. and Andersson et al. [11,13]. These outcomes captured shoulder symptoms, reduced participation, reduced performance, or time loss, rather than relying exclusively on formal medical diagnosis. Other studies used broader outcomes, including coach-recorded time-loss sport-related injuries [12], physician-recorded time-loss shoulder injuries [14], weekly self-reported non-contact or substantial injuries [15], or self-reported injuries during follow-up [16]. This variation in outcome definitions and surveillance methods was considered important when interpreting the prognostic relevance of the screening tests.

### 3.3. Summary of the Main Findings

#### 3.3.1. Overview of Findings

Overall, the findings suggest that shoulder-specific preseason measures showed more consistent associations with shoulder-related outcomes than broader movement-screening tools. In particular, several prospective studies reported that deficits in shoulder strength, altered rotational profile, or scapular control were associated with an increased probability of later shoulder problems in handball players, whereas broader screening batteries such as the 9+ screening battery, the Functional Movement Screen, and the upper quarter Y-balance test generally showed limited prognostic associations with overall injury outcomes [9,10,11,12,13,14,15,16]. The main findings of the included studies are summarized in Table 2.

#### 3.3.2. Shoulder Strength and Muscle Imbalance

Shoulder strength deficits emerged as one of the most recurrent findings across the included studies. In a cohort of youth elite handball athletes, Achenbach et al. [9] found that reduced absolute and normalized external rotation strength, as well as a reduced ER:IR strength ratio, were associated with an increased risk of overuse shoulder injury during the season. Similarly, Clarsen et al. [13] reported that reduced external rotation strength was associated with a greater probability of substantial shoulder problems in elite male handball players. Edouard et al. [14] also observed that a preseason imbalanced shoulder strength profile was associated with an increased risk of subsequent shoulder injury in youth elite female players. Taken together, these findings suggest that shoulder rotator strength, particularly external rotation capacity and strength balance, may have prognostic relevance in handball-specific injury screening.

However, the evidence was not entirely uniform. Andersson et al. [11] did not confirm external rotation weakness as a significant prognostic screening variable in a mixed-sex elite cohort, despite a trend suggesting that a reduced ER:IR ratio might be associated with a higher probability of injury. This inconsistency may reflect differences in sex composition, injury outcome definition, or the specific shoulder variables included in the statistical models.

#### 3.3.3. Range of Motion, Rotational Profile, and Humeral Adaptations

Shoulder range of motion and rotational characteristics were examined in several studies, with mixed results. Clarsen et al. [13] found that reduced total rotational motion was associated with subsequent substantial shoulder problems, supporting the view that restricted rotational capacity may be clinically relevant in elite male handball players. In contrast, Andersson et al. [11] did not confirm reduced total rotation as a significant prognostic variable, but reported that greater internal rotation range of motion was associated with a higher probability of overuse shoulder injury.

Achenbach et al. [9] reported a more nuanced pattern in youth elite players, showing that glenohumeral internal rotation deficit and external rotation gain were associated with overuse shoulder injury in girls only, while these measures were not identified as consistent prognostic associations across the entire cohort. In a later and larger study, Achenbach et al. [10] found that humeral retrotorsion, glenohumeral rotational range of motion, and related adaptation measures were not significantly associated with subsequent overuse injury of the dominant shoulder. These findings suggest that rotational adaptations may not represent consistent prognostic factors across all handball populations, and that their relevance may depend on age, sex, and the exact outcome definition used.

#### 3.3.4. Scapular Control and Dyskinesis

Scapular dyskinesis was assessed in the shoulder-specific cohort studies, but the findings were again mixed. Clarsen et al. [13] found that obvious scapular dyskinesis was associated with an increased probability of substantial shoulder problems. This finding is consistent with the clinical view that altered scapular control may play a role in the development of shoulder symptoms in high-level throwing athletes. By contrast, both Achenbach et al. [9] and Andersson et al. [11] did not find scapular dyskinesis to be significantly associated with subsequent overuse shoulder outcomes. Overall, the available evidence suggests that scapular control may be relevant in some handball cohorts, but current findings are not sufficiently consistent to support a uniform conclusion.

#### 3.3.5. General Movement-Screening Tools

In contrast to the shoulder-specific assessments, the broader movement-screening studies generally did not demonstrate consistent prognostic associations with subsequent injury outcomes. Karlsson et al. [15] found that neither the total score of the 9+ screening battery nor its underlying factor structure was associated with non-contact or substantial injuries in adolescent elite handball players. Slodownik et al. [16] similarly reported that the Functional Movement Screen total score and asymmetry were not significantly associated with future injury, whereas previous injury history was the only variable consistently associated with subsequent injury outcomes. Bauer et al. [12] found that upper quarter Y-balance test performance was not associated with overall injury risk, although one asymmetry parameter was associated with an increased likelihood of lower-body injury. Collectively, these findings suggest limited support for the use of general movement-screening tools as stand-alone prognostic screening tools.

#### 3.3.6. Pattern of Evidence Across Studies

The included studies suggest a distinction between sport-specific shoulder screening and broader movement-screening approaches. The shoulder-focused studies more frequently reported associations with subsequent shoulder-related outcomes, particularly when the outcome of interest was shoulder-specific and the baseline assessment reflected the repetitive throwing demands of handball. In contrast, screening tools designed to capture more general movement quality or upper-quarter balance showed less consistent associations with subsequent injury outcomes, particularly when broader outcome definitions were used. This pattern suggests that screening approaches in handball may demonstrate stronger prognostic associations when the baseline assessment is anatomically and sport-specifically aligned with the injury outcome of interest, as shown in Table 2.

### 3.4. Risk of Bias Within Studies

Overall, the included studies demonstrated moderate methodological quality, with the main concerns relating to confounding, outcome measurement, and statistical analysis and reporting. In general, the shoulder-specific prospective cohort studies were more directly aligned with the anatomical region and injury outcome of interest, whereas the broader movement-screening studies showed greater heterogeneity in screening content and outcome definitions [9,10,11,12,13,14,15,16].

The domains of study participation and prognostic factor measurement were generally acceptable, as most studies clearly described their handball-specific populations and used standardized preseason assessments. However, several studies had relatively small sample sizes, which may have reduced statistical precision and increased the risk of unstable estimates, particularly in Edouard et al. [14] and Slodownik et al. [16].

The most important limitation across studies was confounding, since factors such as previous injury, sex, playing level, training exposure, and playing position were not handled consistently. In addition, outcome measurement varied across studies. Some used validated surveillance tools such as the OSTRC questionnaire or WOSI, whereas others relied on broader or self-reported injury outcomes, increasing the risk of outcome misclassification [9,10,11,13,16].

Overall, greater confidence can be placed in the larger shoulder-specific prospective cohorts, whereas more caution is needed when interpreting findings from the broader movement-screening studies and from studies with smaller samples or less rigorous follow-up. A summary of the risk-of-bias assessment is presented in Table 3.

## 4. Discussion

Pre-season screening is widely used in handball despite limited synthesis regarding which screening approaches demonstrate meaningful prognostic associations with subsequent injury outcomes. Previous reviews in handball have mainly focused on injury epidemiology and shoulder injury risk factors rather than evaluating the prognostic relevance of pre-season screening procedures across different assessment approaches [2,6]. Therefore, the present review aimed to systematically synthesize the available evidence on pre-season screening tests and physical assessments in relation to subsequent injury outcomes in handball players.

The main finding of this review was that shoulder-specific pre-season physical assessments tended to show more consistent prognostic associations with shoulder-related outcomes than broader movement-screening tools. However, these findings should be interpreted cautiously because the evidence base was limited, heterogeneous, and derived exclusively from observational studies [9,10,11,12,13,14,15,16]. Across the included studies, measures such as external rotation strength, shoulder strength imbalance, total rotational motion, and, in some cohorts, scapular dyskinesis or rotational adaptations were associated with subsequent shoulder injury outcomes. Nevertheless, the magnitude and clinical usefulness of these associations varied substantially across studies and were not evaluated through predictive performance metrics. In contrast, broader screening approaches, including the 9+ screening battery, the Functional Movement Screen (FMS), and the upper quarter Y-Balance Test, generally demonstrated limited and inconsistent prognostic associations with broader injury outcomes [9,10,11,12,13,14,15,16].

The interpretation of the findings is strongly influenced by how injury outcomes were defined. In several studies, shoulder overuse injury was not based solely on a medical diagnosis, but on validated patient-reported instruments such as the WOSI or OSTRC questionnaire [9,10,11,13]. This is appropriate in handball because players may continue to train and compete despite shoulder pain, meaning that time-loss definitions alone may underestimate the burden of overuse problems [1,3,13]. However, it also means that the included studies did not measure identical outcomes. Some studies examined associations with shoulder symptoms or substantial shoulder problems [9,10,11,13], whereas others predicted time-loss injuries, non-contact injuries, or broader sport-related injuries [12,14,15,16]. Therefore, the term “prediction” in this review should be interpreted as prognostic association with subsequent injury outcomes, rather than as evidence that screening tests directly prevent injury. Importantly, the presence of a prognostic association does not necessarily indicate clinically useful predictive performance, because statistically significant associations may still demonstrate limited discrimination, accuracy, or practical utility at the individual-athlete level.

This pattern is clinically plausible. Handball places substantial repetitive load on the throwing shoulder, and shoulder overuse problems are a well-recognized issue in both adolescent and elite players [3,6]. It is therefore reasonable that screening variables directly linked to the dominant shoulder would be more likely to show associations with shoulder-related outcomes than general movement-quality tests, particularly when the outcome of interest is also shoulder-specific. In the present review, the studies that tended to show the most consistent associations were those in which the baseline assessment and the injury outcome were anatomically aligned, such as shoulder strength or rotational measures being associated with subsequent shoulder problems [9,13,14].

Among the shoulder-specific findings, external rotation strength and shoulder strength balance appeared to show the most consistent prognostic associations across the included studies. Achenbach et al. [9] reported that reduced external rotation strength and a reduced ER:IR ratio were associated with subsequent overuse shoulder injury outcomes in youth elite players. Similarly, Clarsen et al. [13] found that reduced external rotation strength was associated with an increased probability of substantial shoulder problems in elite male handball players, while Edouard et al. [14] reported that a preseason imbalanced muscular strength profile was associated with a 2.57-fold increased likelihood of subsequent shoulder injury in youth elite female players (RR 2.57, 95% CI 1.60–3.54). Collectively, these findings suggest that measures of rotator cuff capacity and shoulder strength balance may warrant further investigation as preseason screening variables in handball. However, these observed associations should not be interpreted as evidence of causal relationships or as proof of clinically useful predictive performance.

The evidence regarding range of motion and rotational adaptations was less consistent across studies. Clarsen et al. [13] found that reduced total rotational motion was associated with subsequent shoulder problems, whereas Andersson et al. [11] did not consistently support reduced total rotation as a prognostic factor and instead reported that greater internal rotation was associated with an increased probability of overuse shoulder injury (OR 1.16 per 5° increase, 95% CI 1.00–1.34). Achenbach et al. [9] found that glenohumeral internal rotation deficit and external rotation gain were associated with subsequent shoulder injury outcomes in girls only, while Achenbach et al. [10] later reported that humeral retrotorsion and related rotational adaptations were not associated with subsequent overuse shoulder injury outcomes. Collectively, these findings suggest that rotational measures do not demonstrate consistent prognostic associations across all handball populations and outcome definitions. Their observed associations may depend on factors such as age, sex, training history, adaptive changes of the throwing shoulder, and the specific way injury outcomes are defined and monitored. Importantly, these findings should not be interpreted as evidence of causal mechanisms or clinically useful predictive performance. This interpretation is also consistent with previous literature in overhead athletes, where shoulder range-of-motion findings have been heterogeneous and their clinical relevance remains uncertain [4].

The findings for scapular dyskinesis were also inconsistent across studies. Clarsen et al. [13] reported that obvious scapular dyskinesis was associated with an increased probability of substantial shoulder problems, whereas Andersson et al. [11] and Achenbach et al. [9] did not identify scapular dyskinesis as a consistently associated prognostic variable. This inconsistency may reflect methodological differences in the assessment of scapular control, but it may also suggest that scapular dyskinesis does not demonstrate consistent prognostic associations when considered in isolation. Instead, its observed associations may be more relevant when interpreted alongside other shoulder-related characteristics such as external rotation weakness, reduced rotational capacity, or greater throwing exposure. These findings should not be interpreted as evidence that scapular dyskinesis directly contributes to subsequent injury outcomes.

In contrast to the shoulder-specific studies, the findings for general movement-screening tools were less convincing. Karlsson et al. [15] found that neither the total 9+ screening battery score nor its factor structure predicted non-contact or substantial injuries in adolescent elite handball players. Slodownik et al. [16] similarly reported that FMS score and asymmetry were not associated with future injury, while previous injury history was the only variable consistently associated with subsequent injury outcomes. Bauer et al. [12] also found that upper quarter Y-Balance Test performance was not associated with overall injury risk, although one asymmetry threshold was linked to lower-body injury. Taken together, these studies suggest limited support for the use of broad screening tools as stand-alone prognostic screening tools in handball.

There are at least two likely explanations for this finding. First, general movement-screening tools may be too broad to capture the specific mechanisms underlying injury in handball, particularly shoulder overuse problems related to repetitive throwing, force production, and tissue-specific adaptation. Second, the injury outcomes used in these studies were often broader and more heterogeneous, including any time-loss injury, non-contact injury, or substantial injury, which may dilute associations between baseline screening scores and subsequent injury outcomes. This supports the idea that the closer the match between the screening construct and the injury outcome, the stronger the observed prognostic association is likely to be.

From a practical perspective, the present findings suggest that preseason screening in handball should not rely solely on generic movement-screening tools. This does not mean that FMS, 9+ screening, or Y-Balance testing have no value. They may still be useful for general athlete profiling, exercise prescription, rehabilitation planning, or identifying movement limitations. However, based on the available evidence, these tools do not appear to provide strong stand-alone prognostic information for injury risk in handball players. In contrast, shoulder-specific preseason assessments, particularly those focused on external rotation strength, strength balance, and selected aspects of shoulder rotational profile, appear to be more closely associated with shoulder-related outcomes when the objective is to identify players who may be more likely to develop subsequent shoulder problems. For handball coaches and performance staff, preseason screening may be most useful when used to monitor shoulder function longitudinally rather than as a one-time injury identification tool. Screening findings may support individualized load management, targeted strengthening, and clinical follow-up when interpreted within the broader athlete context.

Preseason shoulder screening in handball may benefit from placing greater emphasis on shoulder-specific assessments rather than relying solely on generic movement-screening scores. Based on the current evidence, the preseason screening components most consistently associated with shoulder-related outcomes appear to include external and internal rotation strength, ER:IR strength ratio, side-to-side strength asymmetry, glenohumeral internal and external rotation range of motion, total rotational motion, and observational assessment of scapular dyskinesis [9,10,11,12,13,14]. Hand-held dynamometry may be more feasible for routine team screening than isokinetic testing, although isokinetic assessment can provide more detailed information on concentric and eccentric strength profiles when available [9,13,14]. Screening results should not be interpreted in isolation, but alongside previous injury, current symptoms, sex, age, playing position, training load, and throwing exposure [1,3,6,9,10,11,12,13,14]. Consequently, preseason screening findings should be interpreted as part of a broader multifactorial clinical assessment process rather than as definitive indicators of future injury.

The findings of this review should be interpreted in light of several methodological issues in the included literature. First, the evidence base was heterogeneous with respect to age, sex, competitive level, screening methods, injury definitions, and follow-up procedures. The included populations also varied substantially across adolescent, youth elite, and adult elite players and included male, female, and mixed-sex cohorts, limiting subgroup-specific interpretation. In addition, only eight studies met the eligibility criteria, reflecting the limited availability of prospective research in this field and reducing the confidence with which the findings can be interpreted. Second, several studies had relatively small sample sizes, particularly Edouard et al. [14] and Slodownik et al. [16], which may have reduced statistical precision. Third, important confounders such as previous injury, playing position, training volume, and exposure were not handled consistently across studies. These limitations likely contributed to the variability of findings and reduced the feasibility of quantitative pooling. Similar methodological concerns have also been highlighted in previous reviews of shoulder injury risk factors in handball and overhead athletes [5,6]. Furthermore, none of the included studies was designed to evaluate whether screening-based interventions reduced injury risk. The studies examined whether baseline screening variables were associated with subsequent injury outcomes, but they did not systematically report whether coaches, physiotherapists, or medical staff modified training based on screening results. This is important because targeted strengthening, mobility, and proprioceptive interventions after screening could modify the subsequent injury risk and potentially attenuate the observed association between baseline test results and injury outcomes. Future studies should either control for post-screening interventions or prospectively evaluate screening-linked prevention programmes [9,10,11,12,13,14,15,16]. Another important limitation is that the available evidence was based entirely on heterogeneous observational studies, many of which had moderate risk of bias and relatively small sample sizes [9,10,11,12,13,14,15,16]. Therefore, the findings should not be interpreted as establishing causal relationships or definitive evidence of clinically useful predictive performance for specific screening tests. Rather, the current evidence only suggests possible prognostic associations between some preseason shoulder measures and later injury outcomes. This review also has limitations related to the review process itself. Although the search strategy and Supplementary Materials were revised to improve transparency and reproducibility, formal assessment of reporting bias and certainty of evidence was not performed because of the small number of heterogeneous observational studies and the absence of quantitative synthesis. Furthermore, although the review protocol was prospectively registered in PROSPERO, no major protocol amendments occurred during the review process. Additional limitations relate to the review process itself. Google Scholar was used only as a supplementary source and screening was limited to the first 100 records sorted by relevance. Furthermore, only studies providing extractable handball-specific data were included.

## 5. Conclusions

Overall, the current evidence suggests that sport-specific shoulder screening may demonstrate more consistent prognostic associations with shoulder-related outcomes. Measures related to external rotation strength, shoulder strength imbalance, and selected aspects of shoulder rotational profile appear to be among the preseason screening variables most consistently associated with shoulder-related outcomes. However, the evidence remains heterogeneous and not fully consistent, partly because injury outcomes and surveillance methods varied substantially across studies. Therefore, the findings should be interpreted as evidence of prognostic associations with subsequent injury outcomes rather than proof that screening tests directly prevent injury. Screening results should be interpreted within a broader clinical and training context, alongside previous injury history, training load, playing position, age, sex, and current symptoms, rather than as definitive stand-alone prognostic tools for injury outcomes. Future prospective studies should investigate standardized outcome definitions and evaluate whether screening-based targeted interventions may contribute to reducing injury risk in handball players. At present, the available evidence does not support establishing a gold-standard preseason shoulder screening protocol for handball players.

## Figures and Tables

**Table 1:** Characteristics of included studies.

Study	Country	Design	Participants	Injury Events/Outcomes	Screening/Baseline Assessment	Injury Outcome and Follow-Up	Outcome Definition/Assessment Method	Main Findings
Achenbach et al. (2020) [9]	Germany	Prospective cohort	*n* = 138 youth elite handball players; 70 boys, 68 girls; mean age 14.1 ± 0.8 years	Overuse shoulder symptoms reported prospectively during the 2017–2018 season; exact event count not reported (response rate 63%)	Preseason shoulder screening: passive ROM, isometric ER/IR strength, eccentric ER strength, ER:IR ratio, scapular dyskinesia, throwing velocity	Overuse shoulder injury/symptoms during a 7-month season; repeated questionnaires and WOSI	Overuse shoulder injury/symptoms assessed prospectively using standardized questionnaires and WOSI during the season.	Reduced ER strength and reduced ER:IR ratio were associated with overuse shoulder injury; ER gain and GIRD showed prognostic associations in girls only.
Achenbach et al. (2025) [10]	Germany	Prospective cohort prognosis study	*n* = 258 elite youth handball players; 135 males, 123 females; mean age 14.0 ± 0.8 years	20 athletes reported shoulder overuse injuries over the 2-season follow-up	Preseason humeral retrotorsion, IR, ER, TROM, GIRD, ER gain, TROM gain	Overuse injury of the dominant shoulder across 2 seasons; WOSI-based surveillance	Dominant-shoulder overuse injury assessed prospectively using WOSI-based surveillance over two seasons.	Humeral retrotorsion and ROM-based adaptation measures were not significant prognostic variables of shoulder overuse injury.
Andersson et al. (2017) [11]	Norway	Prospective cohort	*n* = 329 elite handball players; 168 male, 161 female	Overuse shoulder problems monitored prospectively using the OSTRC questionnaire; exact event count not reported	Preseason IR/ER ROM, TROM, isometric ER/IR strength, ER:IR ratio, scapular dyskinesis	Overuse shoulder injury in the dominant shoulder during one season; monthly OSTRC questionnaire	Overuse shoulder injury assessed monthly using the OSTRC Overuse Injury Questionnaire; outcome reflected pain, reduced participation, reduced performance, and severity.	Previously proposed factors were not confirmed; greater IR ROM was associated with a higher probability of overuse shoulder injury.
Bauer et al. (2023) [12]	Germany	Prospective cohort	*n* = 133 adolescent sub-elite handball players; mixed-sex sample	57 sport-related injuries during the competitive season (27 upper-body, 30 lower-body injuries)	Preseason upper quarter Y-balance test (medial, inferolateral, superolateral reach, composite score, asymmetry)	Time-loss sport-related injuries during an ~8-month season; weekly coach-based monitoring	Sport-related time-loss injuries recorded weekly by coaches using an injury report form.	YBT-UQ was generally not associated with overall injury risk; inferolateral asymmetry ≥ 7.75% showed prognostic associations with injury outcomes
Clarsen et al. (2014) [13]	Norway	Prospective cohort	*n* = 206 elite male handball players; mean age 24 years	Average prevalence of shoulder problems: 28%; substantial shoulder problems: 12%; exact cumulative event count not reported	Preseason shoulder IR/ER ROM, TROM, isometric IR/ER/abduction strength, ER:IR ratio, scapular dyskinesis	Substantial shoulder problems during a 30-week season; bi-weekly OSTRC questionnaire	Shoulder problems assessed bi-weekly using the OSTRC Overuse Injury Questionnaire; substantial problems included reduced participation/performance or time loss.	Obvious scapular dyskinesis, reduced TROM, and reduced ER strength were associated with increased shoulder injury risk.
Edouard et al. (2013) [14]	France	Prospective cohort	*n* = 16 youth elite female handball players; mean age 18 ± 1 years	Newly incurred shoulder injuries monitored prospectively during the season; exact event count not reported	Preseason isokinetic IR/ER strength, side-to-side deficits, ER/IR ratios, functional strength ratios, muscular imbalance profile	New time-loss shoulder injury during one season; physician-led follow-up	New time-loss shoulder injuries recorded during the season through physician-led follow-up.	An imbalanced shoulder strength profile was associated with a higher likelihood of subsequent shoulder injury.
Karlsson et al. (2021) [15]	Sweden	Prospective cohort	*n* = 45 adolescent elite handball players; 23 females, 22 males; median age 17 years	Median seasonal substantial injury prevalence: 22%; exact injury-event count not reported	Preseason 9+ screening battery (11 movement-based tests)	Non-contact new injury and substantial injury over 52 weeks; weekly OSTRC questionnaire	Non-contact new injuries and substantial injuries recorded weekly using a web-based OSTRC questionnaire.	Neither the 9+ screening battery total score nor its factor scores predicted injury.
Slodownik et al. (2018) [16]	Poland	Mixed design with 6-month follow-up	*n* = 30 adult male handball players from 1st and 2nd divisions	Injuries assessed retrospectively and after 6-month follow-up; exact event count not reported	Baseline Functional Movement Screen plus prior injury history	Self-reported injuries during the following 6 months	Injuries assessed by self-reported questionnaire during the 6-month follow-up.	Previous injury history predicted future injury; FMS total score and asymmetry did not.

Abbreviations: ROM = range of motion; ER = external rotation; IR = internal rotation; TROM = total rotational motion; GIRD = glenohumeral internal rotation deficit; WOSI = Western Ontario Shoulder Index; OSTRC = Oslo Sports Trauma Research Center; YBT-UQ = upper quarter Y-balance test.

**Table 2:** Main findings of the included studies.

Study	Primary Screening Variable(s)	Injury Outcome	Main Quantitative Finding(s)	Interpretation
Achenbach et al. (2020) [9]	Preseason shoulder ER strength, ER:IR ratio, ER gain, and GIRD	Overuse shoulder injury/symptoms during a 7-month season	Lower absolute ER strength: OR 10.70 (95% CI 1.20–95.60); lower normalised ER strength: OR 1.20 (95% CI 1.00–1.40); lower ER:IR ratio: OR 1.20 (95% CI 1.10–1.50). ER gain > 7.5° and GIRD > 7.5° were risk factors in girls only.	Reduced ER strength and reduced ER:IR ratio predicted shoulder overuse injury; rotational adaptations were relevant only in girls.
Achenbach et al. (2025) [10]	Preseason humeral retrotorsion, IR, ER, TROM, GIRD, ER gain, and TROM gain	Overuse injury of the dominant shoulder across 2 seasons	No significant associations: humeral retrotorsion (*p* = 0.895), ER gain (*p* = 0.129), ER gain > 7.5° (*p* = 0.311), GIRD (*p* = 0.056), GIRD > 7.5° (*p* = 0.311), or TROM gain (*p* = 0.672).	Humeral retrotorsion and ROM-based adaptation measures were not useful prognostic screening variables of shoulder overuse injury.
Andersson et al. (2017) [11]	Preseason IR/ER ROM, TROM, ER/IR strength, ER:IR ratio, and scapular dyskinesis	Overuse shoulder injury in the dominant shoulder during one season	No significant associations for TROM (OR 1.05 per 5° change, 95% CI 0.98–1.13), ER strength (OR 1.05 per 10 N, 95% CI 0.92–1.20), or obvious scapular dyskinesis (OR 1.23, 95% CI 0.25–5.99). Greater IR ROM was associated with higher injury probability: OR 1.16 per 5° (95% CI 1.00–1.34).	Previously proposed prognostic factors were not consistently supported; only greater IR ROM was associated with an increased probability of overuse shoulder injury (OR 1.16 per 5° increase, 95% CI 1.00–1.34).
Bauer et al. (2023) [12]	Preseason YBT-UQ reach scores, composite score, and asymmetry	Time-loss sport-related injuries during an 8-month season	No significant differences in most YBT-UQ measures between injured and non-injured players. Inferolateral reach asymmetry ≥ 7.75% arm length predicted lower-body injury: HR 2.18 (95% CI 1.02–4.68, *p* = 0.045).	YBT-UQ showed limited value for overall injury outcomes; one asymmetry metric was associated with lower-body injury risk.
Clarsen et al. (2014) [13]	Preseason TROM, ER strength, abduction strength, ER:IR ratio, and scapular dyskinesis	Substantial shoulder problems during a 30-week season	Obvious scapular dyskinesis: OR 8.41 (95% CI 1.47–48.10, *p* = 0.02); reduced TROM: OR 0.77 per 5° change (95% CI 0.56–0.99, *p* = 0.046); reduced ER strength: OR 0.71 per 10 Nm change (95% CI 0.44–0.99, *p* = 0.046).	Obvious scapular dyskinesis, reduced TROM, and reduced ER strength were associated with increased shoulder injury risk.
Edouard et al. (2013) [14]	Preseason isokinetic rotator strength, ER/IR ratios, functional ratios, and muscular imbalance profile	New time-loss shoulder injury during one season	Imbalanced muscular strength profile: RR 2.57 (95% CI 1.60–3.54). ER/IR ratio at 240°/s <0.69: RR 2.57 (95% CI 1.60–3.54). IRecc/ERcon ratio > 1.61: RR 2.08 (95% CI 1.18–2.98).	A preseason shoulder strength imbalance profile was associated with a 2.57-fold increased likelihood of subsequent shoulder injury (RR 2.57, 95% CI 1.60–3.54).
Karlsson et al. (2021) [15]	Preseason 9SB total score and factor scores (complex movements, mobility, lower-extremity control)	Non-contact new injury and substantial injury over 52 weeks	AUC values ranged from 0.50 to 0.59. For total 9SB score ≤ 22, OR 1.02 (95% CI 0.29–3.59, *p* = 0.98). No factor score significantly predicted injury.	The 9+ screening battery did not demonstrate meaningful predictive ability for later injury.
Slodownik et al. (2018) [16]	Baseline FMS total score, FMS asymmetry, and previous injury history	Self-reported injuries during the subsequent 6 months	Previous injury history predicted future injury: OR 13.71 (*p* = 0.02). FMS total score ≤ 14: OR 2.80 (*p* = 0.20). FMS asymmetry: OR 0.76 (*p* = 0.72).	Previous injury history, but not FMS score or asymmetry, was associated with an increased likelihood of subsequent injury (OR 13.71, *p* = 0.02).

OR = odds ratio; HR = hazard ratio; RR = relative risk; CI = confidence interval; ER = external rotation; IR = internal rotation; TROM = total rotational motion; GIRD = glenohumeral internal rotation deficit; YBT-UQ = upper quarter Y-balance test; 9SB = 9+ screening battery. Notes: Reported effect estimates are presented as described in the original studies and include adjusted and unadjusted analyses where applicable.

**Table 3:** Risk of bias within studies (QUIPS). Domain-level judgments based on the Quality In Prognosis Studies (QUIPS) tool.

Study	Participation	Attrition	Prognostic Factor Measurement	Outcome Measurement	Confounding	Statistical Analysis/ Reporting	Overall
Achenbach et al., 2020 [9]	Low	Moderate	Low	Low	Moderate	Moderate	Moderate
Achenbach et al., 2025 [10]	Low	Moderate	Low	Low	Moderate	Moderate	Moderate
Andersson et al., 2017 [11]	Low	Moderate	Low	Low	Moderate	Moderate	Moderate
Bauer et al., 2023 [12]	Low	Low	Low	Moderate	Moderate	Moderate	Moderate
Clarsen et al., 2014 [13]	Low	Moderate	Low	Low	Moderate	Moderate	Moderate
Edouard et al., 2013 [14]	Moderate	Low	Low	Moderate	High	High	High
Karlsson et al., 2021 [15]	Low	High	Moderate	Moderate	Moderate	Moderate	Moderate–High
Slodownik et al., 2018 [16]	Moderate	Moderate	Moderate	High	High	High	High

Abbreviations: QUIPS, Quality In Prognosis Studies. Note: Low = low risk of bias; Moderate = some concerns; High = high risk of bias. Domain judgments were based on study design, follow-up completeness, clarity and specificity of prognostic factor and outcome measurement, handling of confounding, and adequacy of statistical reporting.

## Data Availability

No new data were created in this study. Data supporting the reported results are available within the article and its Supplementary Materials.

## Supplementary Materials

The following supporting information can be downloaded at: https://www.mdpi.com/article/10.3390/sports14060234/s1, Supplementary File S1. Full database-specific search strategies.

## Abbreviations

The following abbreviations are used in this manuscript:

CI	Confidence interval
ER	External rotation
ER:IR ratio	External rotation to internal rotation ratio
FMS	Functional Movement Screen
GIRD	Glenohumeral internal rotation deficit
HR	Hazard ratio
IR	Internal rotation
OR	Odds ratio
OSTRC	Oslo Sports Trauma Research Center
PICOS	Population, Intervention/Exposure, Comparator, Outcome, Study design
PRISMA	Preferred Reporting Items for Systematic Reviews and Meta-Analyses
PROSPERO	International Prospective Register of Systematic Reviews
QUIPS	Quality In Prognosis Studies
ROM	Range of motion
RR	Relative risk
TROM	Total rotational motion
WOSI	Western Ontario Shoulder Index
YBT-UQ	Upper quarter Y-Balance Test
9SB	9+ screening battery
