# Supplementary material for: Preseason Screening Tests and Physical Assessments as Predictors of Injury in Handball Players: A Systematic Review
Source: Sports (Basel). 2026 Jun 5;14(6):234. doi: 10.3390/sports14060234 (PMC13306686; doi:10.3390/sports14060234)
Supplement: Supplementary file 1 [file sports-14-00234-s001.zip › sports-4261748-supplementary/Supplementary File 1.pdf]

Supplementary File S1. Full Database-Specific Search Strategies

The complete database-specific search strategies used for this systematic review are presented below in accordance with PRISMA 2020 recommendations.

Databases and Platforms

Database	Platform
PubMed	NCBI
MEDLINE	EBSCOhost
CINAHL	EBSCOhost
Scopus	Elsevier
Google Scholar	Google Scholar

No language or publication date restrictions were applied during the database searches.

Records identified through database searching and supplementary searching were imported into a single spreadsheet and duplicates were removed manually prior to title and abstract screening.

No automation tools or machine-learning screening tools were used during study selection.

Search_Strategy

source | date | search_string_or_method | notes

Search date | 2026-03-14

PubMed | 2026-03-14 | ("handball"[tiab] OR "team handball"[tiab]) AND ("screening test*"[tiab] OR "functional test*"[tiab] OR "movement screening"[tiab] OR "functional movement screen"[tiab] OR "pre-season screening"[tiab] OR "preseason screening"[tiab] OR "pre-participation screening"[tiab] OR "baseline assessment"[tiab]) AND (injury[tiab] OR injuries[tiab] OR "injury risk"[tiab] OR "injury prediction"[tiab] OR predictor*[tiab] OR "risk factor*"[tiab]) | Field tags: tiab

MEDLINE | 2026-03-14 | (handball OR "team handball") AND ("screening test*" OR "functional test*" OR "movement screening" OR "functional movement screen" OR "pre-season screening" OR "preseason screening" OR "pre-participation screening" OR "baseline assessment") AND (injury OR injuries OR "injury risk" OR "injury prediction" OR predictor* OR "risk factor*") | Syntax adapted per platform

CINAHL | 2026-03-14 | (handball OR "team handball") AND ("screening test*" OR "functional test*" OR "movement screening" OR "functional movement screen" OR "pre-season screening" OR "preseason screening" OR "pre-participation screening" OR "baseline assessment") AND (injury OR injuries OR "injury risk" OR "injury prediction" OR predictor* OR "risk factor*") | Syntax adapted per platform

Scopus | 2026-03-14 | TITLE-ABS-KEY (handball OR "team handball") AND TITLE-ABS-KEY ("screening test*" OR "functional test*" OR "movement screening" OR "functional movement screen" OR "pre-season screening" OR "preseason screening" OR "pre-participation screening" OR "baseline assessment") AND TITLE-ABS-KEY (injury OR injuries OR "injury risk" OR "injury prediction" OR predictor* OR "risk factor*") | Field tags: TITLE-ABS-KEY

Google Scholar | 2026-03-14 | (handball OR "team handball") ("screening test*" OR "functional test*" OR "movement screening" OR "functional movement screen" OR "pre-season screening" OR "preseason screening" OR "pre-participation screening" OR "baseline assessment") (injury OR injuries OR "injury risk" OR "injury prediction" OR predictor OR "risk factor") | Sorted by relevance; first 100 screened

Backward citation searching | 2026-03-14 | Reference lists of similar reviews and key eligible handball-specific studies were screened for additional studies. | Additional records found: 3 (Clarsen 2014; Edouard 2013; Andersson 2018)

PRISMA_Counts

PRISMA flow counts (working final for manuscript)

Identification

Database records identified (PubMed+MEDLINE+CINAHL+Scopus) | 66.0

Google Scholar records screened (first 100) | 100.0

Total records identified | 166.0

Duplicates removed | 32.0

Records after duplicates removed (screened) | 134.0

Screening | Exclusion reasons

Records screened (title/abstract) | 134.0 | Stage | Reason | Count

Records excluded (title/abstract) | 123.0 | Title/abstract | Not handball-specific

Reports sought for full text | 11.0 | Title/abstract | Not primary research (review/editorial/chapter/protocol)

Additional reports identified via backward citation searching | 3.0 | Title/abstract | No prospective injury follow-up / not injury prediction

Total full-text reports assessed | 14.0 | Title/abstract | Mixed-sport not separable for handball

Full-text reports excluded, with reasons | 6.0 | Full text | Wrong population / mixed-sport not separable | 2

Studies included in review | 8.0 | Full text | Not primary research (review/chapter) | 1

Full text | Wrong design (no prospective follow-up) | 1

Full text | Non-English full text / insufficient reporting | 1

Full text | Not retrievable / insufficient extraction | 1

TitleAbs_Log

record_id | title | databases | decision | reason_code

1 | A Comparison of Functional Movement Abilities of Elite Male Soccer and Handball Players. | CINAHL, Google Scholar | Excluded at title/abstract (provisional)

2 | A Preventive Model for Muscle Injuries: A Novel Approach based on Learning Algorithms. | CINAHL, Google Scholar, MEDLINE | Excluded at title/abstract (provisional)

3 | A Scoping Review of Factors That Elevate the Risk of Anterior Cruciate Ligament Injury in Elite Male Field Team Sport Athletes | Google Scholar | Excluded at title/abstract (provisional)

4 | A comparison of hamstring muscle activity during different screening tests for non-contact ACL injury | MEDLINE, PubMed | Excluded at title/abstract (provisional)

5 | A prospective evaluation of the Landing Error Scoring System (LESS) as a screening tool for anterior cruciate ligament injury risk | Google Scholar | Excluded at title/abstract (provisional)

6 | A reliable video-based ACL injury screening tool for female team sport athletes | Google Scholar | Excluded at title/abstract (provisional)

7 | A systematic evaluation of field-based screening methods for the assessment of anterior cruciate ligament (ACL) injury risk | Google Scholar | Excluded at title/abstract (provisional)

8 | ACL research retreat VII: an update on anterior cruciate ligament injury risk factor identification, screening, and prevention | Google Scholar | Excluded at title/abstract (provisional)

9 | Acute fatigue impairs neuromuscular activity of anterior cruciate ligament-agonist muscles in female team handball players. | CINAHL, MEDLINE | Excluded at title/abstract (provisional)

10 | Altered medial versus lateral hamstring muscle activity during hop testing in female athletes 1-6 years after anterior cruciate ligament reconstruction | CINAHL, MEDLINE, PubMed | Excluded at title/abstract (provisional)

11 | An Interpretable Machine Learning Framework for Athlete Motor Profiling Using Multi-Domain Field Assessments: A Proof-of-Concept Study | Google Scholar | Excluded at title/abstract (provisional)

12 | An integrated approach to change the outcome part I: neuromuscular screening methods to identify high ACL injury risk athletes | Google Scholar | Excluded at title/abstract (provisional)

13 | An investigation into the use of the Functional Movement Screen as a predictor of injury in CrossFit athletes in the eThekwini municipality | Google Scholar | Excluded at title/abstract (provisional)

14 | Are the functional movement analysis scores of handball players related to athletic parameters? | CINAHL, Google Scholar, MEDLINE | Excluded at title/abstract (provisional)

15 | Assessment of dysfunctional movements and asymmetries in children and adolescents using the Functional Movement Screen—A narrative review | Google Scholar | Excluded at title/abstract (provisional)

16 | Assessment of factors associated with injury risk | Google Scholar | Excluded at title/abstract (provisional)

17 | Assessment of return to play after an acute shoulder injury: protocol for an explorative prospective observational German multicentre study. | MEDLINE | Excluded at title/abstract (provisional)

18 | Association between functional movement screen scores and athletic performance in adolescents: A systematic review | Google Scholar | Excluded at title/abstract (provisional)

19 | Association of pre-season musculoskeletal screening and functional testing with sports injuries in elite female basketball players | Google Scholar | Excluded at title/abstract (provisional)

20 | Association of the Functional Movement Screen™ with match-injury burden in men's community rugby union | Google Scholar | Excluded at title/abstract (provisional)

21 | Association with injury of a Football-specific movement screen | Google Scholar | Excluded at title/abstract (provisional)

22 | Automation to improve efficiency of field expedient injury prediction screening | Google Scholar | Excluded at title/abstract (provisional)

23 | Can injury in major junior hockey players be predicted by a pre-season functional movement screen–a prospective cohort study | Google Scholar | Excluded at title/abstract (provisional)

24 | Changes in functional movement screen scores over a season in collegiate soccer and volleyball athletes | Google Scholar | Excluded at title/abstract (provisional)

25 | Characteristics of functional movement screening testing in elite handball players: indicative data from the 9+ | Google Scholar | Excluded at title/abstract (provisional)

26 | Cluster analysis using physical performance and self-report measures to identify shoulder injury in overhead female athletes. | CINAHL, MEDLINE | Excluded at title/abstract (provisional)

27 | Comparison of Functional Movement Screen, Star Excursion Balance Test, and Physical Fitness in Junior Athletes with Different Sports Injury Risk. | CINAHL, Google Scholar, MEDLINE | Excluded at title/abstract (provisional)

28 | Comparison of drop jumps and sport-specific sidestep cutting: implications for anterior cruciate ligament injury risk screening. | MEDLINE | Excluded at title/abstract (provisional)

29 | Comparison of the Predictive Ability of Balance, as Assessed by Reach and Jump Testing, on Non-Contact Ankle Injury Frequency in High School Athletes | Google Scholar | Excluded at title/abstract (provisional)

30 | Composite functional movement screen score predicts injuries in youth volleyball players: a prospective cohort study | Google Scholar | Excluded at title/abstract (provisional)

31 | Consortium for health and military performance and American College of Sports Medicine Summit: utility of functional movement assessment in identifying musculoskeletal injury risk | Google Scholar | Excluded at title/abstract (provisional)

32 | Contact times of change-of-direction manoeuvres are influenced by age and the type of sports: a novel protocol using the SpeedCourt® system. | CINAHL, MEDLINE | Excluded at title/abstract (provisional)

33 | Correlation between two-dimensional video analysis and subjective assessment in evaluating knee control among elite female team handball players. | MEDLINE, Scopus | Excluded at title/abstract (provisional)

34 | Corrigendum. | MEDLINE | Excluded at title/abstract (provisional)

35 | Corrigendum...Krosshaug T, Steffen K, Kristianslund E, et al. The vertical drop jump is a poor screening test for ACL injuries in female elite soccer and handball players: a prospective cohort study of 710 athletes. Am J Sports Med . 2016;44(4):874-883. | CINAHL | Excluded at title/abstract (provisional)

36 | Detection of Upper Limb Asymmetries in Athletes According to the Stage of the Season-A Longitudinal Study. | MEDLINE | Excluded at title/abstract (provisional)

37 | Development of a screening protocol to identify individuals with dysfunctional breathing | Google Scholar | Excluded at title/abstract (provisional)

38 | Differences and relationship in functional movement screen (FMS™) scores and physical fitness in males and female semi-professional soccer players | Google Scholar | Excluded at title/abstract (provisional)

39 | Differences between functional movement screen and somatotype to young handball and volleyball players | Google Scholar | Excluded at title/abstract (provisional)

40 | Do normative composite scores on the functional movement screen differ across high school, collegiate, and professional athletes? A critical review | Google Scholar | Excluded at title/abstract (provisional)

41 | Drop Jump? Single-Leg Squat? Not if You Aim to Predict Anterior Cruciate Ligament Injury From Real-Time Clinical Assessment: A Prospective Cohort Study Involving 880 Elite Female Athletes. | CINAHL, Google Scholar, MEDLINE | Excluded at title/abstract (provisional)

42 | Effect of COL5A1, GDF5, and PPARA Genes on a Movement Screen and Neuromuscular Performance in Adolescent Team Sport Athletes. | CINAHL, Google Scholar, MEDLINE | Excluded at title/abstract (provisional)

43 | Effect of neuromuscular training on proprioception, balance, muscle strength, and lower limb function in female team handball players. | CINAHL, MEDLINE | Excluded at title/abstract (provisional)

44 | Effects of a preparatory training protocol on the movement and body stability of handball players | Google Scholar | Excluded at title/abstract (provisional)

45 | Efficacy of functional movement screening for predicting injuries in coast guard cadets | Google Scholar | Excluded at title/abstract (provisional)

46 | Entwicklung eines Stufen-Konzeptes für Eingangsuntersuchungen im Basketball und Handball–aktueller Stand im deutschen Leistungssport | Google Scholar | Excluded at title/abstract (provisional)

47 | Evaluation of a two dimensional analysis method as a screening and evaluation tool for anterior cruciate ligament injury | Google Scholar | Excluded at title/abstract (provisional)

48 | Evaluation of the Functional Movement Screen (FMS) in identifying active females who are prone to injury. A systematic review | Google Scholar | Excluded at title/abstract (provisional)

49 | Evaluation of the lower extremity functional test to predict lower limb injuries in professional male footballers | Google Scholar | Excluded at title/abstract (provisional)

50 | Evidence-based concepts for prevention of knee and ACL injuries. 2017 guidelines of the ligament committee of the German Knee Society (DKG) | Google Scholar | Excluded at title/abstract (provisional)

51 | Examining the functional motor screening (FMS) scores of Mobarake Sepahan handball players | Google Scholar | Excluded at title/abstract (provisional)

52 | Exploring the Discriminant Validity of the Modified Arm Care Screen (MACS), Designed for Overhead Athletes, in Detecting Musculoskeletal Risk Factors in the General Population | Google Scholar | Excluded at title/abstract (provisional)

53 | FUNCTIONAL MOVEMENT SCREENING FOR INJURY RISK PREDICTION IN YOUNG HIGH SCHOOL ATHLETES-AN OBSERVATIONAL STUDY | Google Scholar | Excluded at title/abstract (provisional)

54 | Factors influencing the relationship between the functional movement screen and injury risk in sporting populations: a systematic review and meta-analysis | Google Scholar | Excluded at title/abstract (provisional)

55 | Functional Knee Performance Differences in Handball are Depending on Playing Class. | CINAHL, MEDLINE | Excluded at title/abstract (provisional)

56 | Functional Movement Screening: An early detection of the student injury risk in sport class | Google Scholar | Excluded at title/abstract (provisional)

57 | Functional knee stability in non-elite handball: balance and jump performance differ based on players' position. | CINAHL, MEDLINE | Excluded at title/abstract (provisional)

58 | Functional movement screen as a predictor of injury in high school basketball athletes | Google Scholar | Excluded at title/abstract (provisional)

59 | Functional movement screening performance of Brazilian jiu-jitsu athletes from Brazil: differences considering practice time and combat style | Google Scholar | Excluded at title/abstract (provisional)

60 | Is a low Functional Movement Screen score (≤ 14/21) associated with injuries in sport? A systematic review and meta-analysis | Google Scholar | Excluded at title/abstract (provisional)

61 | Is it possible to prevent sports injuries? Review of controlled clinical trials and recommendations for future work. | MEDLINE | Excluded at title/abstract (provisional)

62 | Judging return-to-sport after ACL reconstruction using acyclic as well as cyclic neuromuscular performance tests...German Knee Society (DKG) 8th Annual Congress, November 22-23, 2019, Hamburg, Germany. | CINAHL | Excluded at title/abstract (provisional)

63 | Kiss goodbye to the 'kissing knees': No association between frontal plane inward knee motion and risk of future non-contact ACL injury in elite female athletes | Google Scholar | Excluded at title/abstract (provisional)

64 | Little associations exist between the three commonly used functional screening tests in collegiate athletes | Google Scholar | Excluded at title/abstract (provisional)

65 | Lower Limb Injuries in Women's Handball, Protocols, and Most Common Tests: A Systematic Review. | MEDLINE | Excluded at title/abstract (provisional)

66 | Measures of Knee Capability in Handball Players Differ by Age: A Cross Sectional Study. | CINAHL, MEDLINE | Excluded at title/abstract (provisional)

67 | Movement Screening and Injury Risk | Google Scholar | Excluded at title/abstract (provisional)

68 | No effect of extracorporeal shockwave therapy on patellar tendinopathy in jumping athletes during the competitive season: a randomized clinical trial. | MEDLINE | Excluded at title/abstract (provisional)

69 | Non-contact anterior cruciate ligament and lower extremity injury risk prediction using functional movement screen and knee abduction moment: an epidemiological observation of female intercollegiate athletes | Google Scholar | Excluded at title/abstract (provisional)

70 | Physical conditioning and functional Injury-Screening profile of elite female soccer players: A systematic review | Google Scholar | Excluded at title/abstract (provisional)

71 | Poor validity of functional performance tests to predict knee injury in female soccer players with or without anterior cruciate ligament reconstruction | Google Scholar | Excluded at title/abstract (provisional)

72 | Pre-Activity Screening | Google Scholar | Excluded at title/abstract (provisional)

73 | Pre-Participatory Musculoskeletal Screening in Cricket | Google Scholar | Excluded at title/abstract (provisional)

74 | Pre-season screening of the upper body and trunk in Australian football players: A prospective study | Google Scholar | Excluded at title/abstract (provisional)

75 | Predicting injuries in gymnastics using the functional movement screen | Google Scholar | Excluded at title/abstract (provisional)

76 | Prediction and injury risk based on movement patterns and flexibility in a 6-month prospective study among physically active adults | Google Scholar | Excluded at title/abstract (provisional)

77 | Predictive utility of the functional movement screen and Y-balance test: current evidence and future directions | Google Scholar | Excluded at title/abstract (provisional)

78 | Preliminary insights into the diagnostic accuracy of the modified arm Care screen test for overhead athletes: an on-field tool for injury prevention | Google Scholar | Excluded at title/abstract (provisional)

79 | Preseason shoulder range of motion screening and in-season risk of shoulder and elbow injuries in overhead athletes: systematic review and meta-analysis. | Google Scholar, MEDLINE | Excluded at title/abstract (provisional)

80 | Prevention and screening programs for anterior cruciate ligament injuries in young athletes: a cost-effectiveness analysis | Google Scholar | Excluded at title/abstract (provisional)

81 | Reference values for the closed kinetic chain upper extremity stability test for elite handball players...Third World Congress of Sports Physical Therapy, October 4-5, 2019, Vancouver, British Columbia. | CINAHL | Excluded at title/abstract (provisional)

82 | Relationship between core stability and Functional Movement Screening test in athletes | Google Scholar | Excluded at title/abstract (provisional)

83 | Relationship between functional movement screening score and history of injury | Google Scholar | Excluded at title/abstract (provisional)

84 | Relationship between functional movement screening score and history of injury and identifying the predictive value of the FMS for injury. | CINAHL, Google Scholar, MEDLINE | Excluded at title/abstract (provisional)

85 | Relationship between the history of injury and functional movement screening scores in İran National team wrestlers | Google Scholar | Excluded at title/abstract (provisional)

86 | Relationships between the handball-specific complex test, non-specific field tests and the match performance score in elite professional handball players. | MEDLINE | Excluded at title/abstract (provisional)

87 | Reliability of a field-based drop vertical jump screening test for ACL injury risk assessment | Google Scholar | Excluded at title/abstract (provisional)

88 | Reviewing the Use of Injury Screening Assessments and Identifying Risk of Injury | Google Scholar | Excluded at title/abstract (provisional)

89 | Risiko-Screening im Handballsport–Welche funktionellen Tests sind sinnvoll? | Google Scholar | Excluded at title/abstract (provisional)

90 | Risk factors for musculoskeletal injury in elite pre-professional modern dancers: a prospective cohort prognostic study | Google Scholar | Excluded at title/abstract (provisional)

91 | Screening Tests for ACL Injury: Letter to the Editor...Krosshaug T, Steffen K, Kristianslund E, et al. The vertical drop jump is a poor screening test for ACL injuries in female elite soccer and handball players: a prospective cohort study of 710 athletes. Am J Sports Med. 2016;44(4):874-883. | CINAHL | Excluded at title/abstract (provisional)

92 | Screening and analysis for upper and lower limb injuries in tennis and related sports–A scoping review and recommendations for the LTA. | Google Scholar | Excluded at title/abstract (provisional)

93 | Screening tests for assessing athletes at risk of acl injury or reinjury—a scoping review | Google Scholar | Excluded at title/abstract (provisional)

94 | Season Long Changes in Performance Outcome Measures Using the Functional Preparticipation Examination | Google Scholar | Excluded at title/abstract (provisional)

95 | The Functional Movement Screen and Injury Risk in Sporting Populations: An Evidence-to-Practice Review | Google Scholar | Excluded at title/abstract (provisional)

96 | The Impact of Interaction between Body Posture and Movement Pattern Quality on Injuries in Amateur Athletes | Scopus | Excluded at title/abstract (provisional)

97 | The Relationship between Low Functional Movement Screen Scores, Injury History and the Rate of Injury in Collegiate and Junior Hockey Players | Google Scholar | Excluded at title/abstract (provisional)

98 | The Role of Pre-participation Assessment and Screening in Basketball | Google Scholar | Excluded at title/abstract (provisional)

99 | The Vertical Drop Jump Is a Poor Screening Test for ACL Injuries in Female Elite Soccer and Handball Players. | CINAHL | Excluded at title/abstract (provisional)

100 | The Vertical Drop Jump Is a Poor Screening Test for ACL Injuries: Letter to the Editor...Krosshaug T, Steffen K, Kristianslund E, et al. The vertical drop jump is a poor screening test for ACL injuries in female elite soccer and handball players: a prospective cohort study of 710 athletes. Am J Sports Med. 2016;44(4):874-883. | CINAHL | Excluded at title/abstract (provisional)

101 | The ability of the functional movement screen in predicting injury rates in division I female athletes | Google Scholar | Excluded at title/abstract (provisional)

102 | The association between functional movement screen scores and knee valgus moments during unplanned sidestep cutting in netball | Google Scholar | Excluded at title/abstract (provisional)

103 | The closed kinetic chain upper extremity stability test (CKCUEST) performance in elite team handball players playing with shoulder pain, previous pain, or no pain | Google Scholar | Excluded at title/abstract (provisional)

104 | The drop-jump screening test: difference in lower limb control by gender and effect of neuromuscular training in female athletes | Google Scholar | Excluded at title/abstract (provisional)

105 | The effect of functional movement screen and lower extremity training on hamstring/quadriceps ratio in football players | Google Scholar | Excluded at title/abstract (provisional)

106 | The functional movement screen: a reliability study | Google Scholar | Excluded at title/abstract (provisional)

107 | The functional movement test 9+ is a poor screening test for lower extremity injuries in professional male football players: a 2-year prospective cohort study | Google Scholar | Excluded at title/abstract (provisional)

108 | The impact of sport-specific physical fitness change patterns on lower limb non-contact injury risk in youth female basketball players: a pilot study based on field testing and machine learning | Google Scholar | Excluded at title/abstract (provisional)

109 | The relationship between functional movement screen and isokinetic trunk muscle strength, postural stability and body composition in team sports players | Google Scholar | Excluded at title/abstract (provisional)

110 | The relationship between performance of a single-leg squat and leap landing task: moving towards a netball-specific anterior cruciate ligament (ACL) injury risk screening method | Google Scholar | Excluded at title/abstract (provisional)

111 | The relationship between the functional movement screen and the Y balance test in youth footballers | Google Scholar | Excluded at title/abstract (provisional)

112 | Upper limb functional testing: does age, gender, and sport influence performance? | MEDLINE | Excluded at title/abstract (provisional)

113 | Use of clinical movement screening tests to predict injury in sport | Google Scholar | Excluded at title/abstract (provisional)

114 | Utility of FMS to understand injury incidence in sports: current perspectives | Google Scholar | Excluded at title/abstract (provisional)

115 | Which screening tools can predict injury to the lower extremities in team sports? A systematic review | Google Scholar | Excluded at title/abstract (provisional)

116 | Why screening tests to predict injury do not work—and probably never will…: a critical review | Google Scholar | Excluded at title/abstract (provisional)

117 | [Return to throwing sports after upper extremity injury and overuse : A criteria-based approach using an ulnar collateral ligament injury as an example]. | MEDLINE | Excluded at title/abstract (provisional)

118 | Évaluation du contrôle postural dynamique du membre inférieur dans une population de handballeurs sains de haut niveau. Effet de la latéralité et évolution au cours de la saison. | CINAHL | Excluded at title/abstract (provisional)

119 | مقايسه تعادل ايستا و پويا با آزمون غربالگری عملکردی در پيش بينی بروز آسيب های اندام تحتانی درميان هندباليست هاي مرد دانشگاهي. | CINAHL | Excluded at title/abstract (provisional)

120 | Associations between upper quarter Y-balance test performance and sport-related injuries in adolescent handball players | Google Scholar | Full text assessed – excluded or pending (provisional)

121 | Investigation of the accuracy of functional testing on predicting the severity of lower limb injuries of handball athletes | Google Scholar | Full text assessed – excluded or pending (provisional)

122 | Predicting ACL Injury Using Machine Learning on Data From an Extensive Screening Test Battery of 880 Female Elite Athletes. | CINAHL, MEDLINE | Full text assessed – excluded or pending (provisional)

123 | THE RELATIONSHIP AMONG FUNCTIONAL MOVEMENT SCREEN MEASUREMENTS, INJURY HISTORY AND BODY COMPOSITION IN ELITE MALE HANDBALL PLAYERS | Google Scholar | Full text assessed – excluded or pending (provisional)

124 | The Vertical Drop Jump Is a Poor Screening Test for ACL Injuries in Female Elite Soccer and Handball Players: A Prospective Cohort Study of 710 Athletes. | Google Scholar, MEDLINE | Full text assessed – excluded or pending (provisional)

125 | The role of pre-participation assessment (PPA) and screening in handball | Google Scholar | Full text assessed – excluded or pending (provisional)

126 | [Risk screening in handball: Which functional tests are useful?] | MEDLINE | Full text assessed – excluded or pending (provisional)

127 | Decreased external rotation strength is a risk factor for overuse shoulder injury in youth elite handball athletes. | CINAHL, MEDLINE | Included (provisional)

128 | Functional Movement Screen™ and history of injury in the assessment of potential risk of injury among team handball players. | CINAHL, MEDLINE, Scopus | Included (provisional)

129 | Handball and movement screening - can non-contact injuries be predicted in adolescent elite handball players? A 1-year prospective cohort study. | CINAHL, Google Scholar, MEDLINE | Included (provisional)

130 | Increased humeral retrotorsion is not a risk factor for overuse injury of the throwing shoulder in elite youth handball athletes. | MEDLINE | Included (provisional)

131 | Modifying the functional movement screen deep squat test: The effect of foot and arm positional variations | Google Scholar (manual) | Excluded at title/abstract (final) | TA1

132 | Intra and inter-rater reliability of screening for movement impairments: movement control tests from the foundation matrix | Google Scholar (manual) | Excluded at title/abstract (final) | TA3

133 | Risk factors for lower limb injury in female team field and court sports: a systematic review, meta-analysis, and best evidence synthesis | Google Scholar (manual) | Excluded at title/abstract (final) | TA2

134 | Knowledge, use, and perceptions of functional movement screening tools among certified athletic trainers | Google Scholar (manual) | Excluded at title/abstract (final) | TA1

FullText_Decisions

ft_id | title | final_decision | reason_code | source_url

1 | Functional Movement Screen™ and history of injury in the assessment of potential risk of injury among team handball players | Include | https://pubmed.ncbi.nlm.nih.gov/28967241/

2 | Handball and movement screening - can non-contact injuries be predicted in adolescent elite handball players? A 1-year prospective cohort study | Include | https://pubmed.ncbi.nlm.nih.gov/31663797/

3 | Decreased external rotation strength is a risk factor for overuse shoulder injury in youth elite handball athletes | Include | https://pubmed.ncbi.nlm.nih.gov/30927025/

4 | Increased humeral retrotorsion is not a risk factor for overuse injury of the throwing shoulder in elite youth handball athletes | Include | https://pubmed.ncbi.nlm.nih.gov/39216695/

5 | Associations between upper quarter Y-balance test performance and sport-related injuries in adolescent handball players | Include | https://pubmed.ncbi.nlm.nih.gov/37077424/

6 | [Risk screening in handball: Which functional tests are useful?] | Exclude | FT5

7 | The Vertical Drop Jump Is a Poor Screening Test for ACL Injuries in Female Elite Soccer and Handball Players: A Prospective Cohort Study of 710 Athletes | Exclude | FT2

8 | Predicting ACL Injury Using Machine Learning on Data From an Extensive Screening Test Battery of 880 Female Elite Athletes | Exclude | FT2

9 | The role of pre-participation assessment (PPA) and screening in handball | Exclude | FT1

10 | Investigation of the accuracy of functional testing on predicting the severity of lower limb injuries of handball athletes | Exclude | FT7

11 | THE RELATIONSHIP AMONG FUNCTIONAL MOVEMENT SCREEN MEASUREMENTS, INJURY HISTORY AND BODY COMPOSITION IN ELITE MALE HANDBALL PLAYERS | Exclude | FT3

12 | Reduced glenohumeral rotation, external rotation weakness and scapular dyskinesis are risk factors for shoulder injuries among elite male handball players: a prospective cohort study | Include | https://pubmed.ncbi.nlm.nih.gov/24948083/

13 | Shoulder strength imbalances as injury risk in handball | Include | https://pubmed.ncbi.nlm.nih.gov/23444085/

14 | Risk factors for overuse shoulder injuries in a mixed-sex cohort of 329 elite handball players: previous findings could not be confirmed | Include | https://pubmed.ncbi.nlm.nih.gov/28784621/

Reason_Codes

reason_code | stage | description

TA1 | Title/abstract | Not handball-specific population

TA2 | Title/abstract | Not primary research (review/editorial/chapter/protocol)

TA3 | Title/abstract | No prospective injury follow-up / not injury prediction

TA4 | Title/abstract | Screening exposure not relevant (e.g., biomechanics validation only)

TA5 | Title/abstract | Mixed-sport data not extractable for handball

TA6 | Title/abstract | Conference abstract / letter / corrigendum

TA7 | Title/abstract | Other / unclear from abstract

FT1 | Full text | Not primary research (review/chapter)

FT2 | Full text | Wrong population (not handball or mixed-sport not separable)

FT3 | Full text | Wrong design (no prospective follow-up)

FT4 | Full text | Wrong exposure (not pre-season screening/assessment)

FT5 | Full text | Non-English full text / insufficient report

FT6 | Full text | Outcome not injury / not reportable

FT7 | Full text | Not retrievable / insufficient reporting for extraction

Google Scholar Search Procedure

Google Scholar results were sorted by relevance, and the first 100 records were screened for potential eligibility, consistent with common systematic review practice.

Backward Citation Searching

Backward citation searching identified 3 additional records/reports, of which 1 met the inclusion criteria and was included in the review.

Consistency Note

The study by Andersson et al. was consistently referred to as Andersson et al. 2017 throughout the manuscript and supplementary materials.
